# A Systematic Narrative Review of Idiopathic Granulomatous Mastitis: Evidence, Management Challenges, and Recurrence Patterns

**DOI:** 10.7759/cureus.111598

**Published:** 2026-06-27

**Authors:** Vanessa Msosa, Casten Chikumbutso, Hanafih U Nkhata, Shadreck Mtambo, Varun Patnam

**Affiliations:** 1 Breast Surgery, Swansea Bay Breast Unit, Swansea, GBR; 2 Surgery, Kamuzu Central Hospital, Lilongwe, MWI; 3 Surgery, Manchester University NHS Foundation Trust, Manchester, GBR

**Keywords:** breast inflammation, diagnosis, idiopathic granulomatous mastitis, recurrence, treatment outcomes

## Abstract

Idiopathic granulomatous mastitis (IGM), also known as granulomatous lobular mastitis, is a rare, benign, chronic inflammatory breast disease that primarily affects women of reproductive age and frequently mimics breast carcinoma clinically and radiologically, making diagnosis challenging. Its aetiology remains poorly understood, and treatment is not standardised. This systematic narrative review was conducted according to the Preferred Reporting Items for Systematic Reviews and Meta-Analyses (PRISMA) guidelines and included English-language studies published between January 2009 and March 2026 identified through PubMed, Ovid MEDLINE, Scopus, Google Scholar, and Cumulative Index to Nursing and Allied Health Literature (CINAHL). Studies addressing the aetiology, clinical features, diagnosis, management, or outcomes of IGM were eligible, and data were extracted using a standardised Microsoft Excel form (Microsoft Corp., Redmond, WA, USA). A total of 271 studies were included, with publication trends demonstrating increasing research activity over time and the highest volume of studies published between 2023 and 2025. Most studies were retrospective observational studies (29.7%; n = 81) or case reports (22.3%; n = 61), while prospective studies (8.8%; n = 24) and interventional trials (4.8%; n = 13) were limited. Thematic analysis showed that treatment and therapeutics were the most frequently studied areas (31%; n = 120), followed by diagnosis and diagnostic methods (25%; n = 95). Recurring findings included diagnostic uncertainty, a lack of standardised treatment protocols, variable recurrence risk, and inconsistent predictors of treatment escalation, while common limitations included small sample sizes, retrospective study designs, single-centre data, and methodological heterogeneity. Overall, IGM remains a diagnostically difficult condition with uncertain aetiology and heterogeneous management; despite growing research interest, the overall level of evidence remains low, highlighting the need for multicentre prospective studies and standardised diagnostic and treatment guidelines to improve evidence-based care and patient outcomes.

## Introduction and background

Idiopathic granulomatous mastitis (IGM), also referred to as granulomatous lobular mastitis (GLM), is a rare, benign, chronic inflammatory breast disease that predominantly affects women of reproductive age, most commonly within 2-6 years postpartum [1-3]. First described by Kessler and Wolloch in 1972 [4], the condition continues to pose significant diagnostic and therapeutic challenges. Although non-malignant, IGM frequently mimics breast carcinoma both clinically and radiologically [5-7], often leading to misdiagnosis and, in some cases, unnecessary invasive interventions [1,2,8].

IGM is a diagnosis of exclusion [9]. Clinically, it typically presents as a unilateral breast mass associated with pain, erythema, nipple discharge, and axillary lymphadenopathy [5,10]. Advanced disease may manifest with abscess formation, sinus tract development, ulceration, and nipple retraction [11,12]. Systemic and extramammary manifestations, including erythema nodosum, arthritis, and episcleritis, have also been reported [13,14]. Histopathologically, the disease is characterised by non-caseating granulomatous inflammation localised to breast lobules [15]. Despite extensive investigation, its aetiology remains poorly understood, with proposed mechanisms including autoimmune processes, hormonal influences, infectious agents, and environmental or lifestyle factors such as smoking and obesity [14-16]. Furthermore, imaging modalities such as ultrasonography, mammography, and magnetic resonance imaging (MRI) lack pathognomonic features, further complicating diagnosis [17].

Epidemiological data on IGM remain limited and variable across regions [18]. It is estimated to have a low annual prevalence, with higher reporting rates in Asia and the Mediterranean region [18,19]. The disease predominantly affects parous women around the age of 30, while occurrence in men and nulliparous women is rare [20-22].

Management of IGM remains heterogeneous, with no universally accepted treatment guidelines [23]. Corticosteroids are widely considered first-line therapy due to their anti-inflammatory effects [24]; however, this knowledge gap has contributed to methodological heterogeneity in research, limiting comparability across studies and hindering robust evidence synthesis and prognostic research. Their use is associated with significant relapse rates and potential adverse effects, particularly with prolonged administration [25,26]. While conservative management and imaging surveillance may be appropriate in selected cases [27], a subset of patients require escalation of therapy, including immunosuppressive agents such as methotrexate or surgical intervention [28-30]. Emerging and adjunctive treatment modalities, including minimally invasive techniques like ozone therapy and alternative herbal therapies, have also been explored, though evidence remains limited and inconsistent [31-34]. Overall, IGM is characterised by a relapsing course, with reported recurrence rates of approximately 17%, necessitating prolonged follow-up and individualised management strategies [35,36].

Several studies have attempted to identify clinical, radiological, and pathological factors associated with disease severity, recurrence, and the need for escalation of therapy. However, findings remain inconsistent, and no reliable predictors of treatment response or progression have been established. Moreover, existing reviews have largely focused on treatment modalities, with limited emphasis on determinants of step-up therapy and associated outcomes. This knowledge gap has contributed to methodological heterogeneity, limiting comparability across studies and hindering robust evidence synthesis and prognostic research. Given these gaps, a comprehensive synthesis of current evidence is required to better inform clinical decision-making and optimise patient care.

The available literature is highly heterogeneous with respect to study design, patient populations, treatment approaches, outcome definitions, and follow-up periods. Because of this substantial methodological and clinical heterogeneity, quantitative pooling through formal meta-analysis was not considered appropriate, and a systematic narrative review was undertaken to provide a structured synthesis of the available evidence while accommodating this heterogeneity.

This systematic narrative review aims to evaluate clinical, imaging, and pathological factors predictive of step-up therapy in patients with IGM and to assess the association between treatment strategies and patient outcomes.

## Review

**Figure 1:**
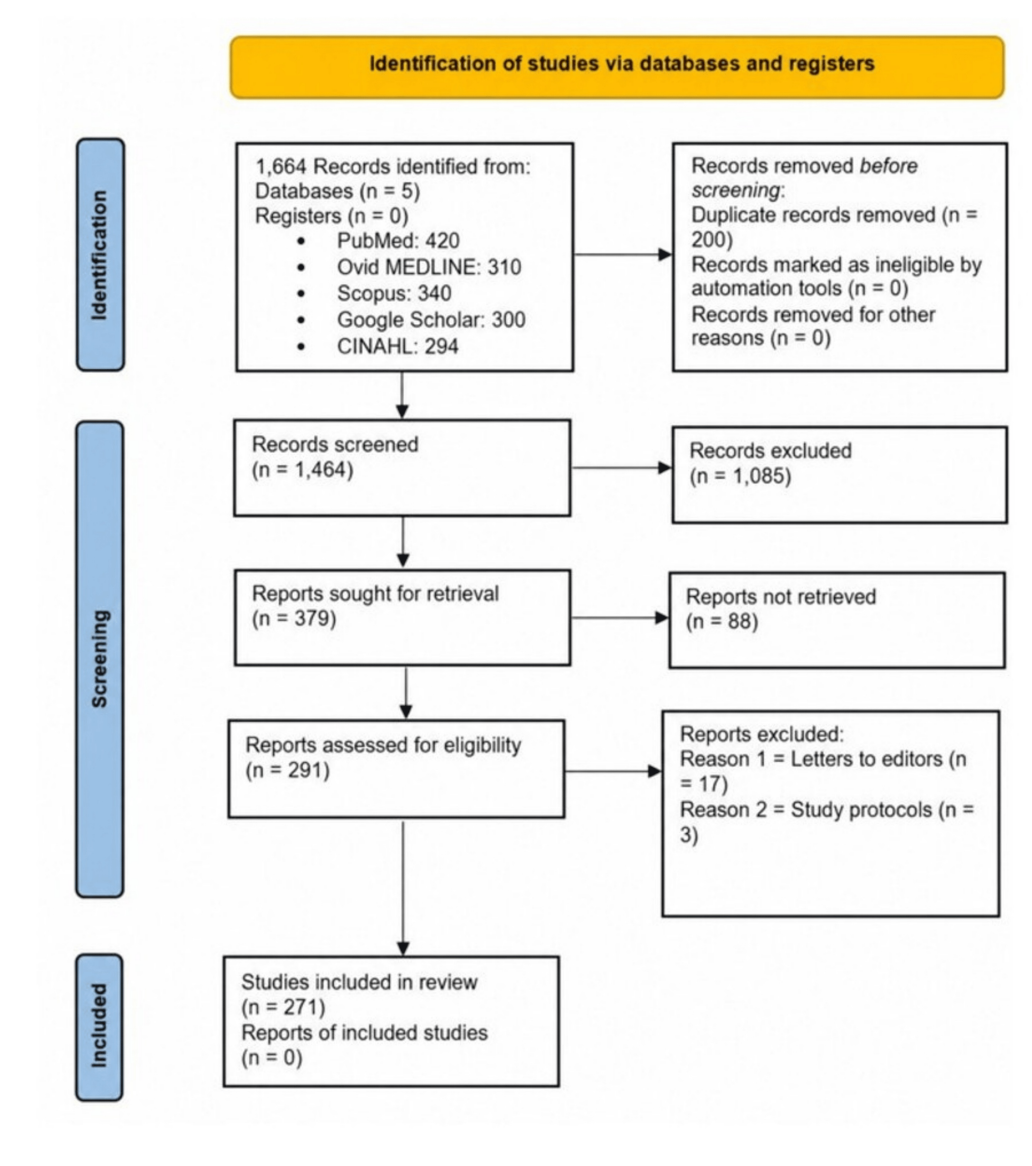
PRISMA flow diagram CINAHL: Cumulative Index to Nursing and Allied Health Literature; PRISMA: Preferred Reporting Items for Systematic Reviews and Meta-Analyses

This systematic review was conducted in accordance with the Preferred Reporting Items for Systematic Reviews and Meta-Analyses (PRISMA) guidelines, as shown in Figure 1. The review included studies on IGM and GLM published between January 2009 and March 2026. Inclusion criteria were studies published in English, all study designs (systematic reviews, cross-sectional studies, retrospective and prospective studies, cohort studies, case series, and case reports), and studies focusing on aetiology, clinical features, diagnosis, management, and/or treatment outcomes. Exclusion criteria were studies published prior to 2009, non-English publications, and studies without fully accessible text.

A comprehensive literature search was conducted across these electronic databases: PubMed, Ovid MEDLINE, Scopus, Google Scholar, and Cumulative Index to Nursing and Allied Health Literature (CINAHL). The library search catalogue combined Medical Subject Headings (MeSH) and free-text terms using Boolean algebra: (granulomatous mastitis) OR (idiopathic granulomatous mastitis) OR (mastitis granulomatous) OR ((granulomatous mastitis) OR (breast inflammation)) OR (granulomatus mastitis) AND (diagnosis)) OR ("granulomatous mastitis") OR ((granulomatous mastitis)) OR (idiopathic granulomatous mastitis)) OR ((granulomatous mastitis) AND (diagnosis)) OR ((chronic breast inflammation) AND (granulomatous)) OR (mastitis granulomatous) OR (granulomatous mastitis).

The search identified 1,664 records, including 420 from PubMed, 310 from Ovid MEDLINE, 340 from Scopus, 300 from Google Scholar, and 294 from CINAHL. After the removal of 200 duplicate records, 1,464 records remained for title and abstract screening. Of these, 1,085 records were excluded, leaving 379 reports sought for retrieval. Eighty-eight reports could not be retrieved, and 291 full-text articles were assessed for eligibility. Twenty articles were excluded following full-text review, including 17 letters to the editor and three study protocols.

A total of 271 studies met the eligibility criteria and were included in the final analysis. Four independent reviewers screened titles and abstracts. One external validator was involved. Discrepancies were resolved through a general consensus among the four authors of this paper. Data was extracted using a standardised data collection form in Microsoft Excel (Microsoft Corp., Redmond, WA, USA). The following variables were collected: study characteristics (authors, year of publication, study design), study focus, key findings, and study limitations.

Coding was performed independently by the review team using a standardised data extraction form. Studies addressing multiple topics were assigned to the theme most closely aligned with the primary objective of the study. Following coding, related categories were grouped into broader themes to facilitate narrative synthesis and the interpretation of the evidence. The thematic categories included the following: treatment and therapeutics, diagnosis and diagnostic methods, clinical features and presentation, treatment outcomes and recurrence, aetiology and pathogenesis, risk factors and associations, imaging and radiology, biomarkers and laboratory studies, management strategies, and miscellaneous topics. Any discrepancies in thematic assignment were discussed among reviewers and resolved through consensus, with oversight from an independent external validator. This structured approach enabled the consistent classification of studies and transparent identification of major research trends, evidence gaps, and recurring findings across the literature.

Ethical approval was not required, as this study was based on an analysis of previously published data.

Results

A total of 271 studies spanning from January 2009 to March 2026 were included in this review.

Publication Year Distribution

There was a clear increase in research activity over time as shown in Table 1. Early studies between 2009 and 2015 accounted for less than 5% of publications, reflecting an initial exploratory phase. This was followed by a gradual rise between 2016 and 2019 (5-15%) and a more pronounced increase from 2020 to 2022 (16-30%). The highest volume of publications was observed between 2023 and 2026 (>30%), indicating peak research interest in recent years.

**Table 1:** Publication year distribution

Period	Approx. frequency	Interpretation
2009-2015	<5%	Early research phase
2016-2019	5-15%	Growing interest
2020-2022	16-30%	Rapid increase
2023-2026	>30%	Peak research activity

Study Design Distribution

**Figure 2:**
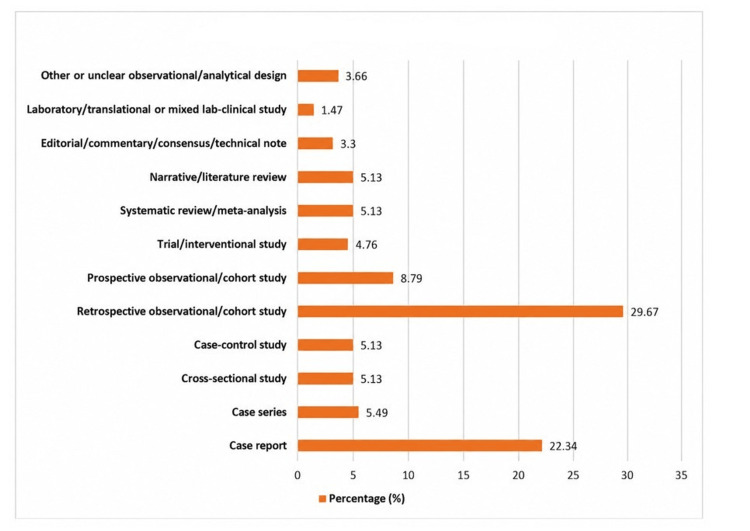
Study design distribution (with percentages)

The included studies were predominantly retrospective cohort/observational in nature as shown in Figure 2.

Retrospective cohort/observational studies constituted the largest proportion of the literature (29.7%; n = 81), followed by case reports (22.3%; n = 61). Prospective studies accounted for 8.8% (n = 24), while interventional trials were limited (4.8%; n = 13). Systematic reviews (n = 14; 5.1%), narrative reviews (n = 14; 5.1%), and cross-sectional or case-control studies (n = 14 each; 5.1%) each represented approximately 5% of the literature. Laboratory-based studies were the least represented (1.5%; n = 4), while mixed-methods studies accounted for 3.3% (n = 9).

Thematic analysis demonstrated that the majority of studies focused on treatment-related topics as shown in Table 2. Treatment and therapeutics (31%;n =120) and diagnostic approaches (25%; n = 95) were the most frequently investigated themes, whereas relatively few studies addressed management strategies or guideline development (1%; n = 5).

**Table 2:** Thematic analysis frequency table

Theme	Frequency (number of studies)	Percentage (%)
Treatment and therapeutics	120	31%
Diagnosis and diagnostic methods	95	25%
Clinical features/presentation	55	14%
Treatment outcomes and recurrence	45	12%
Aetiology/pathogenesis	30	8%
Risk factors and associations	20	5%
Imaging/radiology	10	3%
Biomarkers/lab studies	8	2%
Management strategies	5	1%
Miscellaneous	2	<1%

Common methodological limitations were identified across the included studies as shown in Table 3. Small sample size was the most frequently reported limitation (~40%; n = 120), followed by retrospective design (~23%; n = 70) and limited generalisability (~15%;n = 45). Lack of standardisation in diagnostic and treatment approaches was also noted in approximately 8% (n = 25) of studies.

**Table 3:** Thematic analysis of limitations

Code	Limitation theme	Frequency (number of studies)	Percentage (%)
1	Small sample size	120	~40%
2	Retrospective design	70	~23%
3	Single-centre/lack of generalisability	45	~15%
4	Lack of standardisation (treatment/diagnosis)	25	~8%
5	Short follow-up/loss to follow-up	20	~7%
6	Bias (selection, recall, observational)	15	~5%
7	Lack of control/comparison group	12	~4%
8	Data limitations (missing variables, poor records)	10	~3%
9	Heterogeneity of studies	8	~3%
10	Limited methodology (descriptive only/no prospective validation)	6	~2%

Discussion

Publication Year Distribution

IGM was first described in 1972. Our literature review demonstrates that very little research was conducted in the early years following its initial description.

To enhance interpretability, publication frequency was categorised using predefined proportional thresholds: minimal activity (<5%), emerging activity (5-15%), substantial activity (16-30%), and dominant activity (>30%). These thresholds were adapted from established approaches in descriptive epidemiology and data interpretation, where categorisation of proportions into ordinal groups is commonly used to improve the interpretability of distributions. Future analysis may benefit from calculating the exact percentages of publications within specific time periods to support proportion-based comparisons.

Study Design Distribution

Retrospective studies (29.7%; n = 81) and case reports (22.3%; n = 61) dominate the literature, resulting in a body of evidence largely based on lower levels of evidence. The absence of standardised treatment protocols further limits the development of high-quality evidence and contributes to heterogeneous management approaches, including variable corticosteroid regimens, immunosuppressive use, and surgical indications. Inconsistencies in outcome definitions, follow-up duration, and recurrence reporting also reduce the reliability and reproducibility of findings, limiting the development of evidence-based guidelines.

This pattern likely reflects both the rarity and complexity of IGM. Early evidence generation commonly relies on case reports, case series, and retrospective studies, while the low prevalence of the disease and prolonged follow-up required for recurrence assessment limit the feasibility of large randomised controlled trials and meta-analyses. Additionally, the concentration of reported cases in Asia and the Mediterranean, often within low- and middle-income settings, may further restrict the ability to conduct resource-intensive prospective studies [18,19].

Table 4 summarises the key themes identified across the included studies, including treatment effectiveness, recurrence, diagnostic challenges, imaging findings, aetiology, pathogenesis, risk factors, biomarkers, and multidisciplinary care.

**Table 4:** Thematic organisation of key findings AGR: albumin-to-globulin ratio; NLR: neutrophil-to-lymphocyte ratio, miR: molecular interaction; PTEN: phosphatase and tensin homologue; TCM: traditional Chinese medicine; IGM: idiopathic granulomatous mastitis; GLM: granulomatous lobular mastitis; MRI: magnetic resonance imaging; ADC: apparent diffusion coefficient; PET/CT: positron emission tomography/computed tomography; FNAC: fine needle aspiration cytology; NETs: neutrophil extracellular traps; CRP: C-reactive protein; EN: erythema nodosum

Theme	What the findings show
1. Treatment effectiveness	Steroids, methotrexate, intralesional steroids, topical steroids, bromocriptine, azathioprine, TCM, tinosporin, ozone, and multimodal therapy were all reported as useful in at least some patients. Many papers suggest medical therapy can reduce surgery needs.
2. Comparison of treatment modalities	Several studies compared oral steroids vs intralesional steroids, medical vs surgical treatment, conservative vs combined treatment, and TCM-based vs conventional therapy. A repeated pattern is that no single treatment is universally superior and treatment should be individualised.
3. Recurrence and relapse	Many studies focused on recurrence predictors. Reported recurrence-related factors included smoking, abscesses, bilateral disease, hormonal factors, pregnancy/lactation history, oral contraceptive use, residual lesions, skin lesions, and some biomarker profiles.
4. Diagnosis remains difficult	A major recurring message is that IGM/GLM often mimics breast cancer, tuberculosis, abscess, or other mastitis and that histopathology is the diagnostic gold standard. Imaging is useful, but often nonspecific.
5. Imaging and radiology	Ultrasound, MRI, diffusion-weighted MRI, elastography, ADC maps, PET/CT, FNAC, and radiomics were studied. Imaging helps assessment and differential diagnosis, but many findings stress that imaging alone is not definitive.
6. Clinicopathological features	Many findings describe characteristic pathological patterns such as lobulocentric granulomatous inflammation, neutrophils, lipid vacuoles, abscess formation, and features of cystic neutrophilic granulomatous mastitis.
7. Aetiology and pathogenesis	Aetiology remains unclear, but several mechanisms recur: autoimmunity, immune dysregulation, prolactin/hormonal imbalance, *Corynebacterium* involvement, cytokines, NETs, macrophages, and possible genetic susceptibility.
8. Microbiology and *Corynebacterium*	*Corynebacterium kroppenstedtii* and related species were repeatedly implicated. Some studies support targeted antibiotic therapy and specialised microbiology methods.
9. Risk factors and associations	Frequently reported associations include pregnancy, breastfeeding, parity, smoking, hyperprolactinemia, nipple inversion, hormonal disorders, hypothyroidism, obesity, and contraceptive use.
10. Biomarkers and prognostic markers	IL-6, IL-18, IL-33, CRP, AGR, NLR, mRNA, miR-21/PTEN, serum biomarkers, and staging systems were explored for severity, recurrence, prognosis, and differential diagnosis.
11. Special populations and uncommon presentations	Some findings focused on pregnancy, lactation, male IGM, bilateral disease, COVID-19 era cases, association with EN/episcleritis/arthritis, and coexistence with breast cancer.
12. Need for multidisciplinary and standardised care	Many papers emphasised lack of consensus, controversial management, and the need for multidisciplinary, individualised, and standardised algorithms.

Aetiology, Pathogenesis, Risk Factors, and Biomarkers

There remain considerable gaps in understanding the aetiology of IGM, including potential causative, aggravating, and recurrence-related factors. It would be valuable to investigate whether variations exist across different populations and geographical regions (e.g., Asia, Africa, and Western populations).

Although several studies have attempted to identify clinical, radiological, and pathological predictors of disease severity, recurrence, and treatment escalation, findings remain inconsistent. No reliable predictors of treatment response or disease progression have been established.

Moreover, existing reviews have largely focused on treatment modalities, with limited emphasis on determinants of step-up therapy and associated outcomes. Given these gaps, a comprehensive synthesis of current evidence is necessary to better inform clinical decision-making and optimise patient care.

Diagnostic Challenges

In recent years, granulomatous mastitis has increasingly become a diagnosis of exclusion. Advances in breast imaging and laboratory diagnostics in high-income settings have enabled the more accurate identification of infectious and other secondary causes of mastitis. This trend is now becoming relevant in developing regions, including parts of Asia and Africa, where diagnostic capabilities are improving.

IGM often mimics breast malignancy and presents a significant diagnostic dilemma. This is particularly important in Asian and African settings, where distinguishing granulomatous mastitis from breast cancer is critical to avoid misdiagnosis and inappropriate management.

In low-resource settings, breast ultrasound remains the primary imaging modality due to its accessibility and cost-effectiveness, whereas more advanced imaging techniques such as MRI and computed tomography (CT) are less commonly available. Microbiological and histopathological investigations play a crucial role in diagnosis.

Treatment Effectiveness

Management of IGM continues to be highly variable, reflecting the absence of standardised treatment guidelines and the ongoing uncertainty regarding optimal therapeutic strategies. Corticosteroids remain the most commonly employed first-line therapy because of their anti-inflammatory effects; however, their use is tempered by notable relapse rates and the risk of adverse effects, particularly with prolonged systemic administration. Evidence from Alper et al. [25] further highlights this variability, demonstrating that both systemic and locally administered corticosteroids can be effective, with local therapy offering similar outcomes while potentially minimising systemic toxicity. This supports the notion that no single corticosteroid regimen has emerged as clearly superior.

While conservative management and imaging surveillance may be appropriate in selected cases, a subset of patients requires escalation of therapy. Immunosuppressive agents, particularly methotrexate, have emerged as a useful steroid-sparing option. Findings from Kehribar et al. [36] demonstrate that methotrexate can achieve favorable outcomes, particularly in recurrent or steroid-resistant disease.

Emerging and adjunctive therapies, including minimally invasive approaches and alternative treatments, have also been explored, although the evidence remains limited and inconsistent.

Imaging Findings

In parallel, advances in imaging underscore the evolving and non-uniform nature of IGM management. Findings from Kapetas et al. [26] suggest that acoustic radiation force impulse (ARFI) imaging may enhance diagnostic confidence by improving differentiation between IGM and malignant lesions, as well as offering a tool for disease monitoring. The integration of such modalities into clinical practice varies across centres, further contributing to differences in management approaches. Collectively, these factors reinforce that IGM treatment remains heterogeneous, requiring individualised decision-making based on clinical presentation, available expertise, and patient-specific considerations.

Recurrence

IGM is characterised by a relapsing and often unpredictable clinical course, with reported recurrence rates of approximately 17%, necessitating not only individualised management strategies but also prolonged and vigilant follow-up. Evidence from Fattahi et al. [35] indicates that recurrence is influenced by a range of factors, including treatment modality, disease extent, and patient-specific characteristics.

Importantly, recurrences may occur months to years after apparent clinical resolution, reinforcing the need for extended surveillance even in patients who initially respond well to therapy. However, the potential for relapse despite treatment further underscores the importance of continued clinical and radiological follow-up to monitor for disease reactivation and guide timely intervention.

Overall, the relapsing nature of IGM, coupled with variable treatment responses, necessitates a long-term, structured follow-up strategy to ensure the early detection of recurrence and optimisation of patient outcomes.

Multidisciplinary Care and Future Research Priorities

A substantial proportion of studies focus on treatment strategies, reflecting the absence of a gold-standard therapy. Current management approaches often resemble a trial-and-error process. Significant uncertainties remain regarding the following: which patients are most likely to respond to corticosteroids, predictors of recurrence following treatment, comparative effectiveness of different steroid regimens (e.g., injectable vs systemic), and the influence of patient-specific and treatment-related factors on outcomes. These gaps highlight the need for more robust and standardised research.

Limitations

This review has several limitations. Many of the included studies had small sample sizes, which may limit the generalisability of the findings. There was also a lack of standardised data collection methods across studies, contributing to heterogeneity in the reported outcomes. In addition, the potential for selection bias cannot be excluded, particularly given the predominance of retrospective designs. Finally, some studies contained missing or incomplete data, which may affect the overall reliability of the analysis.

## Conclusions

IGM is a rare and diagnostically challenging inflammatory breast disease with an uncertain aetiology. Although research interest has increased substantially in recent years, the available evidence remains limited and is predominantly derived from retrospective studies and case reports. Proposed mechanisms include autoimmune, hormonal, infectious, and environmental factors, but no definitive cause has been established.

While numerous studies have evaluated treatment approaches, evidence regarding predictors of treatment escalation, recurrence, and disease progression remains limited and inconsistent. No reliable clinical, radiological, or pathological predictors have been identified to guide treatment selection or risk stratification. Consequently, management remains heterogeneous, with corticosteroids representing the most commonly used therapy, but no clear consensus regarding optimal treatment. Future multicentre prospective studies using standardised outcome measures and treatment protocols are needed to establish robust prognostic factors and support the development of evidence-based management guidelines.
